# VDAC1 is essential for neurite maintenance and the inhibition of its oligomerization protects spinal cord from demyelination and facilitates locomotor function recovery after spinal cord injury

**DOI:** 10.1038/s41598-019-50506-4

**Published:** 2019-10-01

**Authors:** Vera Paschon, Beatriz Cintra Morena, Felipe Fernandes Correia, Giovanna Rossi Beltrame, Gustavo Bispo dos Santos, Alexandre Fogaça Cristante, Alexandre Hiroaki Kihara

**Affiliations:** 1 Centro de Matemática, Computação e Cognição, Universidade Federal do ABC, São Bernardo do Campo, SP Brazil; 2 Instituto de Ortopedia e Traumatologia, Faculdade de Medicina da Universidade de São Paulo, São Paulo, SP Brazil

**Keywords:** Cell death in the nervous system, Regeneration and repair in the nervous system

## Abstract

During the progression of the neurodegenerative process, mitochondria participates in several intercellular signaling pathways. Voltage-dependent anion-selective channel 1 (VDAC1) is a mitochondrial porin involved in the cellular metabolism and apoptosis intrinsic pathway in many neuropathological processes. In spinal cord injury (SCI), after the primary cell death, a secondary response that comprises the release of pro-inflammatory molecules triggers apoptosis, inflammation, and demyelination, often leading to the loss of motor functions. Here, we investigated the functional role of VDAC1 in the neurodegeneration triggered by SCI. We first determined that *in vitro* targeted ablation of VDAC1 by specific morpholino antisense nucleotides (MOs) clearly promotes neurite retraction, whereas a pharmacological blocker of VDAC1 oligomerization (4, 4′-diisothiocyanatostilbene-2, 2′-disulfonic acid, DIDS), does not cause this effect. We next determined that, after SCI, VDAC1 undergoes conformational changes, including oligomerization and N-terminal exposition, which are important steps in the triggering of apoptotic signaling. Considering this, we investigated the effects of DIDS *in vivo* application after SCI. Interestingly, blockade of VDAC1 oligomerization decreases the number of apoptotic cells without interfering in the neuroinflammatory response. DIDS attenuates the massive oligodendrocyte cell death, subserving undisputable motor function recovery. Taken together, our results suggest that the prevention of VDAC1 oligomerization might be beneficial for the clinical treatment of SCI.

## Introduction

Spinal cord injury (SCI) can affect mobility and sensibility, first mainly due to axonal rupture and secondly, due to tissue environment toxicity and the consequent inflammatory response. Nervous tissue has a very limited ability to regrow, thus, the neuronal damage is often permanent. As a severe clinical condition, the mechanical injury of the spinal cord has important consequences, including autonomic deregulation and chronic pain^1–3^.

As mentioned, the progression of a neuronal lesion can be separated into two phases^4–6^. The primary injury occurs immediately after the trauma and leads to neuronal/oligodendrocyte cell death and axonal shear^6^, while the secondary injury comprises progressive activation waves of pathophysiological mechanisms, including microvascular perfusion changes^7^, inflammation^8^, lipid peroxidation, free radicals generation, cell death by apoptosis/necrosis^9,10^, homeostasis disruption^11,12^, and demyelination^13^. In fact, the secondary injury often alters the metabolism of intact axonal tracts, mainly due to extensive oligodendrocyte cell death. All these events induce massive necrosis in axons, evolving to demyelination^14^, and this process has been considered the main impediment to the functional recovery after SCI^15,16^.

These events subsequently lead to the alteration of mitochondrial permeability through the interaction of external proteins, such as B-cell lymphoma 2 (Bcl-2) protein family and α-synuclein, with mitochondrial transmembrane proteins^17–19^, disturbing its metabolism and triggering the apoptosis intrinsic pathway^20,21^. Activation of this pathway starts when pro-apoptotic signals overcome anti-apoptotic signals, leading to the release of proteins to the cytosol^22^, such as cytochrome-C (Cyto-C)^23^, which can interact with apoptotic protease-activating factor 1 (Apaf-1), forming the apoptosome. This complex interacts with pro-caspases 9 and 3, promoting cell apoptosis^24^.

One of the most important mitochondrial transmembrane class of porin family is the voltage-dependent anion-selective channels, composed by VDAC1, VDAC2, and VDAC3. As described in previous studies, VDAC1 is the most abundant isoform in the central nervous system^25^. However, this porin was not described in the spinal cord so far. VDAC1 allows the passage of several metabolites, such as pyruvate, succinate, malate, reduced nicotinamide adenine dinucleotide (NADH), reactive oxygen species (ROS)^21,26,27^, and ATP^28^, in addition to specific ions, including Na^+^, K^+^, Ca^2+^ and Cl^−^^26,29,30^. 3D-structures of recombinant VDAC1 obtained by X-ray crystallography revealed that its structure is composed of 19 β-strands arranged as a barrel^31^ and can assemble into dimers, trimers, tetramers, and megapores, facilitating the release of apoptogenic proteins, such as Cyto-c, apoptosis-inducing factor (AIF), and Smac/DIABLO, from the intermembrane space into the cytosol^32,33^. Moreover, VDAC1 can also associate with cytosolic, mitochondrial and cytoskeletal proteins and other membrane channels^34–37^. Additionally, this porin also has an N-terminal region consisting of 25 amino acids, that lies inside the channel pore and can be exposed to the cytoplasm, due to voltage gating^38,39^. Therefore, both processes – assembling into complex arrangements and modulating channel conductance by N-terminal residues – modulate the apoptosis pathway by the control of Cyto-C releasing, as well as impacting other cell death mechanisms^32,33,40,41^.

The 4, 4′-diisothiocyanatostilbene-2, 2′-disulfonic acid (DIDS) is a well-known chloride channel blocker that directly interacts with and inhibits VDAC1 oligomerization^42,43^. Although DIDS cannot be considered as a specific VDAC1 inhibitor, given its interaction with various transport systems^44–48^, it is often described as regulating intrinsic apoptosis mediated by the mitochondria^49^.

Considering all these information, we first realized that VDAC1 is important for neurite maintenance *in vitro*. Next, we determined that VDAC1 immunofluorescence (IF) increases following SCI. Furthermore, we examined the beneficial impact of the blockade of VDAC1 oligomerization with DIDS on initial secondary injury response, which decreased oligodendrocyte cell death, and improved axonal density, as well as allowed the motor function recovery in rats.

## Results

### Reduced VDAC1 expression levels impairs cell metabolism and neurite outgrowth *in vitro*

**Figure 1:**
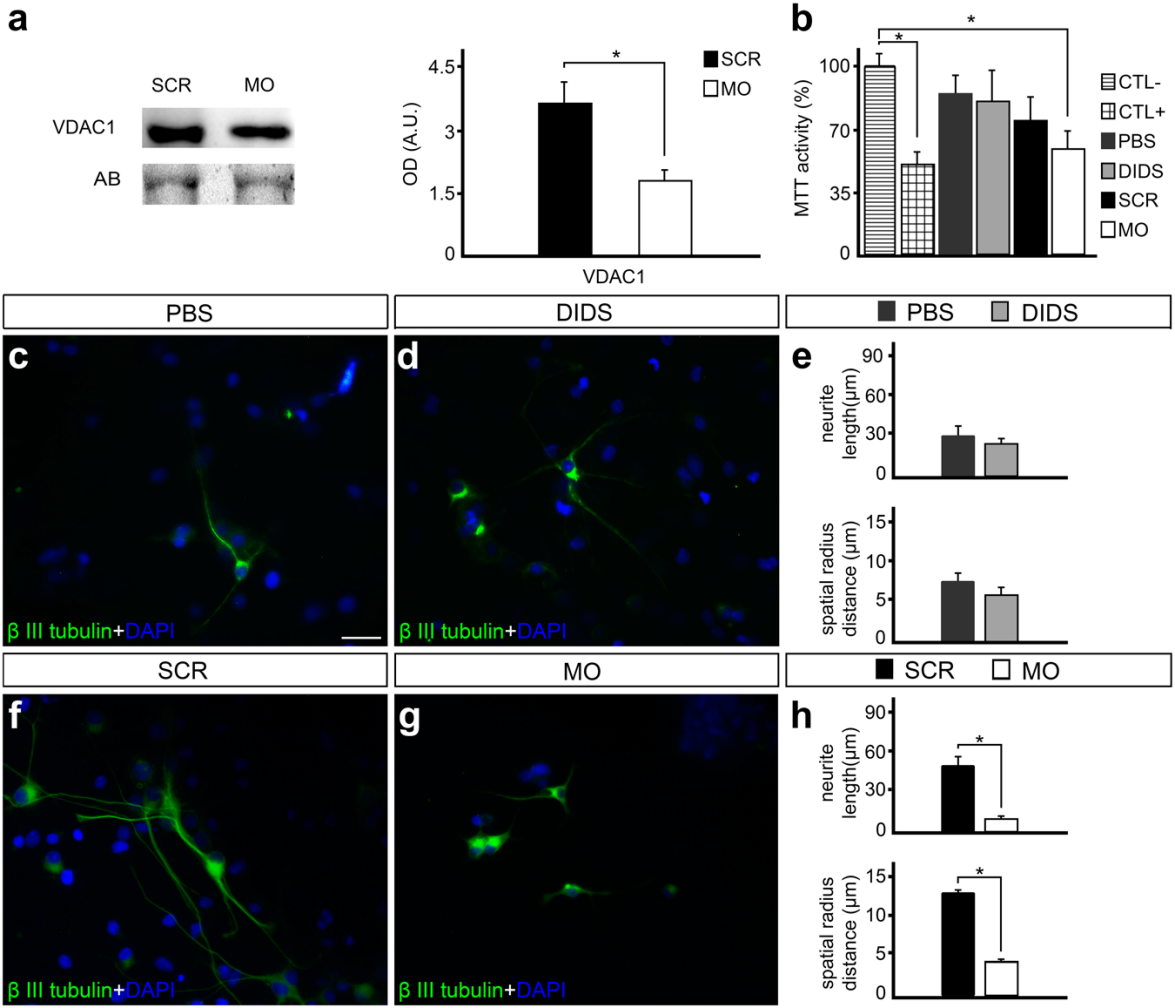
Impact of voltage-dependent anion-selective channel 1 (VDAC1) ablation by morpholino antisense nucleotides (MOs) and pharmacological blockade by 4,4′-diisothiocyanatostilbene-2,2′-disulfonic acid (DIDS) on primary spinal cord cell cultures. (**a**) VDAC1 and representative amido black (AB) bands in spinal cord primary cell cultures treated with scramble (SCR) or VDAC1 MOs and the quantification of respective optical densities of VDAC1. (**b**) Graph of 3-(4,5-dimethyl-2-thiazolyl)-2,5-diphenyltetrazolium bromide (MTT) reductase activity after subjecting to specific conditions, including negative control (CTL−), positive control (CTL+), phosphate-buffered saline (PBS), DIDS, SRC, and MO treatments. Representative images of cell cultures treated with (**c**) PBS or (**d**) 25uM DIDS in immunofluorescence experiments using anti – β III tubulin (green) and counterstained with 4′-6-diamino-2-phenylindole (DAPI, blue). (**e**) Graph indicating the neurite length and radius of the neuronal branch after PBS and DIDS treatment. Representative images of cell cultures treated with (**f**) SCR oligonucleotides or (**g**) VDAC1 MO in immunofluorescence experiments using anti – β III tubulin (green) and counterstained with DAPI (blue). (**h**) Graph showing the neurite length and radius of the neuronal branch after SCR and MO treatments. Bars represent standard error of mean. Scale bar: 50 μm. *P < 0.05.

Experiments with spinal cord primary cell cultures using VDAC1 morpholino antisense nucleotides (MOs) were carried out to determine the role of this porin in neuronal cell survival and neurite outgrowth. As expected, western blot (WB) experiments revealed that treatment of the cells with VDAC1 MOs (10 μM) significantly decreased VDAC1 protein levels in 14 day cell cultures after 48 hours of exposition (55.2 ± 15.2%, P < 0.05, Fig. 1a). We were also able to determine that reduced VDAC1  protein levels affected mitochondrial metabolism as observed by MTT reductase activity assay after MO treatment (54.9 ± 11.4%, *P* < 0.05) when compared to negative control. As expected, positive control also shows decreased MTT reductase activity (48.0 ± 10.7%, *P* < 0.05). On the other hand, changes in MTT reductase activity were not detected after DIDS treatment (Fig. 1b).

Next, we examined the consequences on neuronal morphology. We were not able to detect significant changes in neurite length and spatial radius distance after DIDS treatment (Fig. 1c–e). On the other hand, when compared to controls provided by the exposition to scramble MO, the selective ablation of VDAC1 promoted reduction in both neurite length (52.13 ± 6.36 vs. 10.22 ± 1.76, P < 0.01) and spatial radius distance (13.32 ± 1.33 vs. 4.68 ± 0.98, P < 0.01) (Fig. 1f–h).

### VDAC1 accumulates in different spinal cord cells

**Figure 2:**
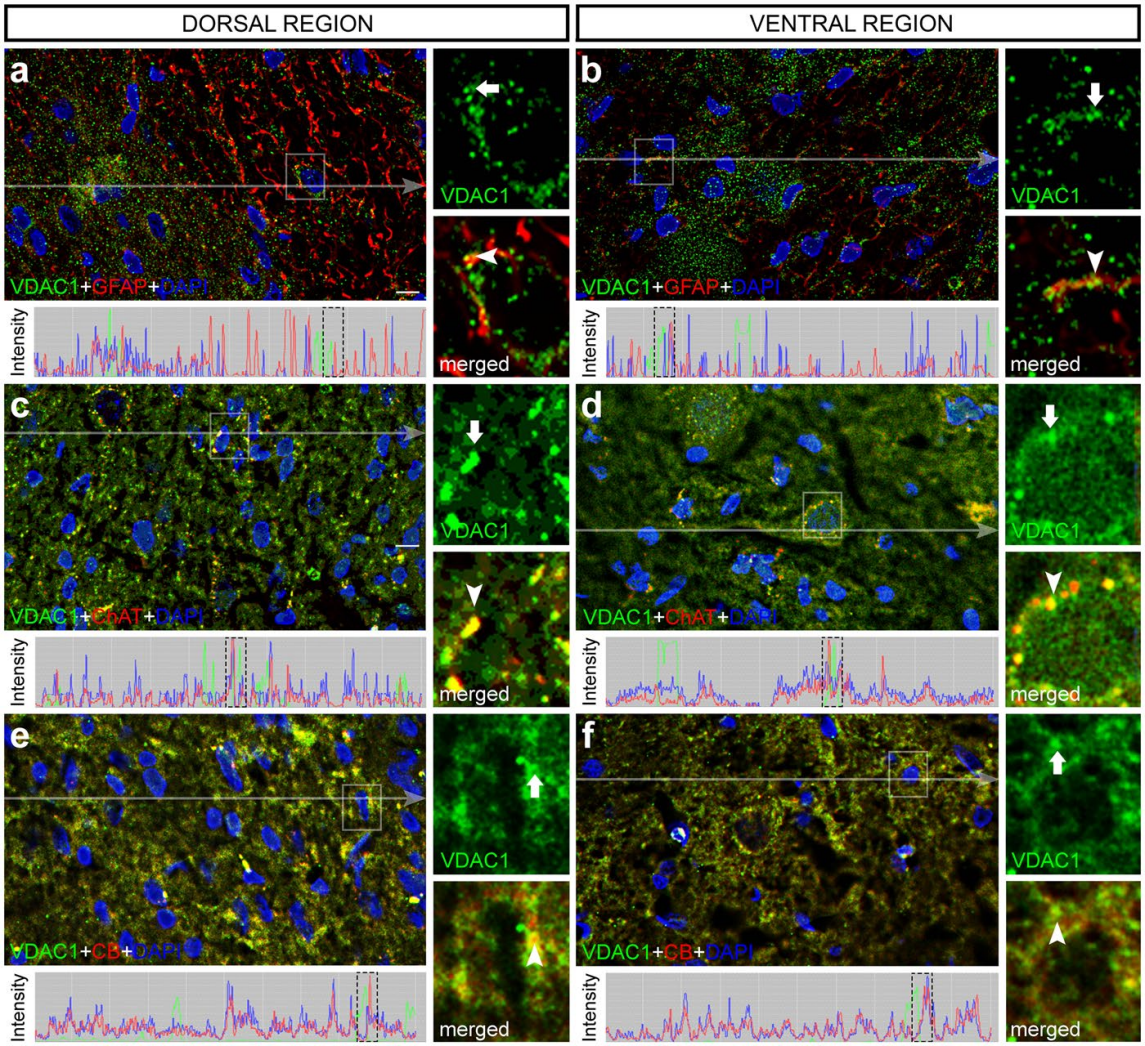
Double labeling immunofluorescence analysis of voltage-dependent anion-selective channel 1 (VDAC1, green) with specific cell markers (red) counterstained with 4′-6-diamino-2-phenylindole (DAPI, blue) in transverse sections of rat spinal cord. Dorsal region of rat spinal cord stained with VDAC1 and (**a**) glial fibrillary acidic protein (GFAP), (**c**) choline acetyltransferase (ChAT), and (**e**) calbindin (CB), counterstained with DAPI. Ventral region of rat spinal cord stained with VDAC1 and (**b**) GFAP, (**d**) ChAT, and (**f**) CB, counterstained with DAPI. Pixel profile analysis revealed colocalization of green and red signals. High magnification view of the selected area confirmed the colocalization of green (arrow) and red (arrowhead) signals. Scale bars: 50 μm.

Double labeling experiments with anti-glial fibrillary acidic protein (GFAP) showed that VDAC1 accumulates in GFAP-positive cells (Fig. 2a,b). Moreover, employing other cell markers as anti-choline acetyltransferase (ChAT) and anti-calbindin (CB) we were able to observe that VDAC1 labeling is present in motor neurons and in   interneurons as well (Fig. 2c–f).

### VDAC1 immunofluorescence (IF) increased 24 h after spinal cord injury

**Figure 3:**
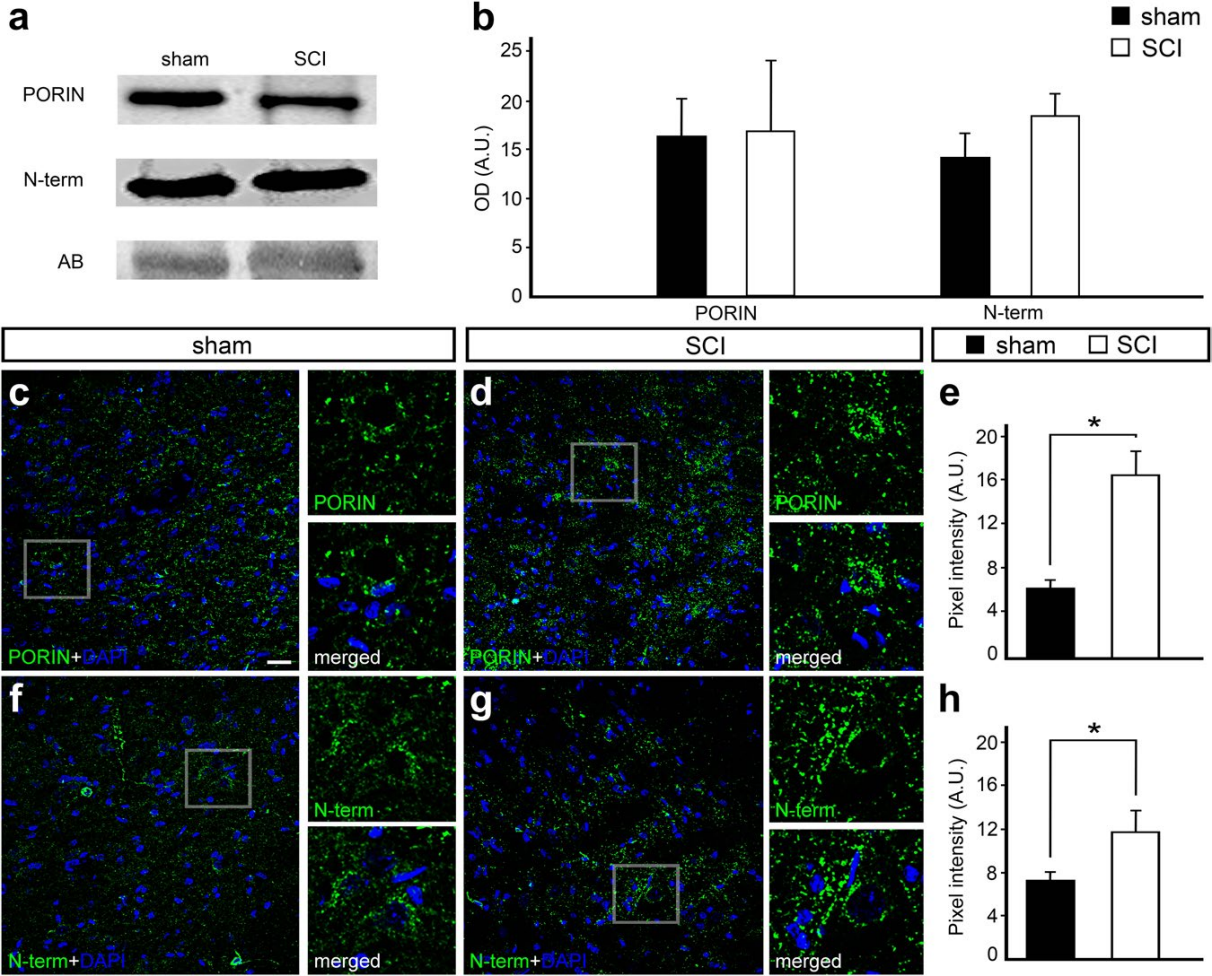
Western blot and immunofluorescence analysis of voltage-dependent anion-selective channel 1 (VDAC1) 24 hours after spinal cord injury (SCI) counterstained with 4′-6-diamino-2-phenylindole (DAPI). (**a**) Representative western blot bands using anti-VDAC1 PORIN, anti-VDAC1 N-term in the spinal cord samples of sham controls and SCI animals. Amido black bands were used as loading controls. (**b**) Quantification of optical densities from the bands obtained using anti-PORIN and anti-N-term in spinal cord samples from sham controls and SCI animals. Representative image of the ventral region of the spinal cord from (**c**) sham controls and (**d**) SCI animals stained with anti-PORIN (green) and counterstained with DAPI (blue), with high magnification view of the respective selected area. (**e**) Graph indicating the pixel intensity of anti-PORIN labeling in sham controls and SCI animals. Representative image of the ventral region of the spinal cord from (**f**) sham controls and (**g**) SCI animals stained with anti-N-term (green) and counterstained with DAPI (blue), with high magnification view of the respective selected area. (**h**) Graph indicating the pixel intensity of N-term labeling in sham controls and SCI animals. Bars represent standard error of the mean. Scale bar: 50 μm. *P < 0.01.

In order to detect if SCI changes VDAC1 protein levels, WB experiments with anti-VDAC-PORIN (VDAC-P), which detects most VDAC1 channels, revealed no changes after injury (Fig. 3a,b). However, it should be noted that WB experiments were performed in the spinal cord encompassing both dorsal and ventral regions and the focus of the primary injury in surrounding areas as well. We next focused on changes in protein distribution and IF intensity, which was analyzed by single IF assay using anti-VDAC1-PORIN (anti-PORIN) and anti-VDAC1-N-term (anti-N-term) antibodies. N-term antibody detects the N-terminal end, which is hidden in the VDAC1 internal pore in physiological conditions and exposed in response to mitochondrial stress. We were thus able to detect differences in IF experiments depending on the analyzed region. The injury was performed at the dorsal region, where no differences were observed comparing sham with SCI (data not shown). On the other hand, when compared to sham animals, anti-PORIN IF signal increased significantly after injury (6.48 ± 0.38 vs. 16.91 ± 2.03, respectively, P < 0.01) in the ventral horn (Fig. 3c–e). Similarly, when compared to controls, anti-N-terminal IF signals also significantly increased after injury (7.37 ± 0.45 vs. 11.78 ± 1.80, respectively, *P* < 0.01) in the ventral horn (Fig. 3f–h).

### VDAC1 pharmacological blockade did not alter initial neuroinflammatory response

**Figure 4:**
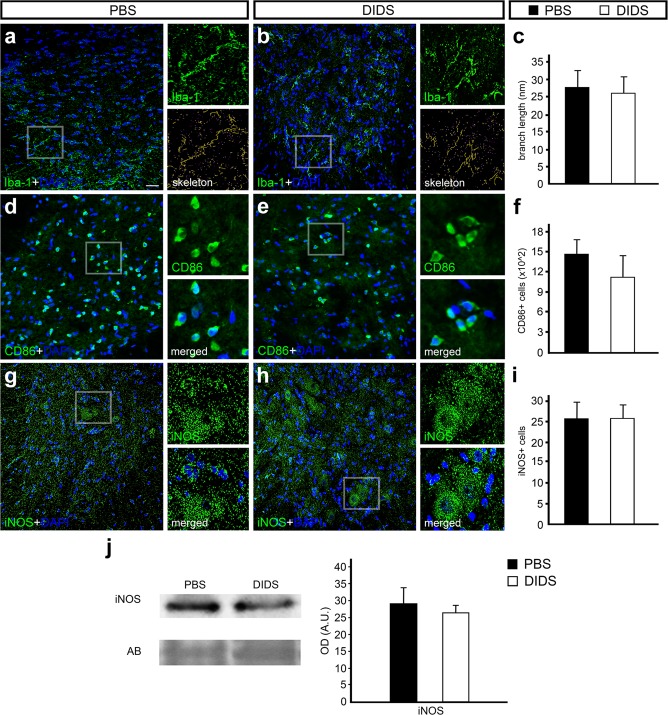
Immunofluorescence and Western blot analysis of ionized calcium binding adaptor molecule 1 (Iba-1), cluster of differentiation 86 (CD86), and inducible nitric oxide synthase (iNOS) counterstained with 4′-6-diamino-2-phenylindole (DAPI) in transverse sections of rat spinal cord treated with phosphate-buffered saline (PBS) or 4,4′-diisothiocyanatostilbene-2,2′-disulfonic acid (DIDS) 24 h after injury. Representative images from the ventral region of (**a**) PBS- and (**b**) DIDS-treated groups stained with Iba-1 (green) and counterstained with DAPI (blue), with high magnification view of the respective selected area and tagged skeleton showing its voxels. (**c**) Graph indicating the average branch length of Iba-1-positive cells in PBS- and DIDS-treated groups. Representative images from the ventral region of (**d**) PBS- and (**e**) DIDS-treated groups stained with CD86 (green) and counterstained with DAPI (blue), with high magnification view of the respective selected area. (**f**) Graph indicating the number of CD86-positive cells in PBS- and DIDS-treated groups. Representative images from the ventral region of (**g**) PBS- and (**h**) DIDS-treated group stained with iNOS (green) and counterstained with DAPI (blue), with high magnification view of the respective selected area. (**i**) Graph indicating the number of iNOS-positive cells in PBS- and DIDS-treated groups. (**j**) Representative iNOS and amido black (AB) bands of spinal cord samples from PBS- and DIDS-treated animals. Quantification of the optical density of iNOS bands, comparing PBS- and DIDS-treated animals. Bars represent standard error of the mean. Scale bar: 50 μm.

Several events occur after SCI, including cellular metabolism alteration and microglial activation, resulting in the proliferation and activation of microglial cells. These processes result in the increase of inducible nitric oxide synthase (iNOS), whose activation depends on mitochondria and which can be harmful to neuronal tissues [34]. Single IF for ionized calcium binding adaptor molecule 1 (Iba-1) revealed that both animal groups have a similar number of Iba-1-positive cells (data not shown). Using a software for image transformation in the skeleton, we were able to measure microglial branching, which was not significantly different comparing PBS with DIDS groups (Fig. 4a–c). We also performed IF using CD86 antibody, a marker of activated microglia, and we were not able to determine changes in the number of CD86-positive cells comparing the PBS and DIDS groups (Fig. 4d–f). Finally, to verify the iNOS protein levels and distribution, we performed WB and IF assays. Both WB and IF for iNOS revealed no significant differences in iNOS protein levels and in the number of positive cells in the spinal cord of animals treated with PBS vs. DIDS after mechanical injury (Fig. 4g–j).

### VDAC1 blockade decreases oligodendrocyte cell death triggered by SCI

**Figure 5:**
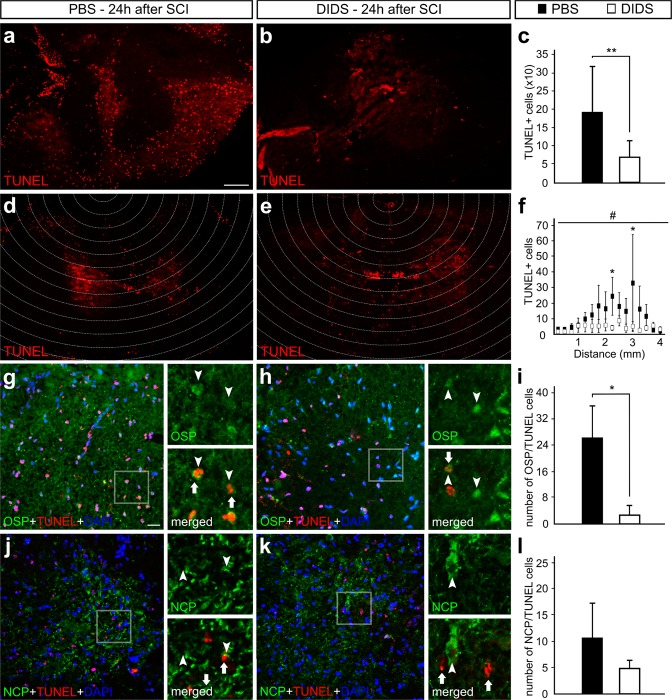
Terminal deoxynucleotidyl transferase dUTP nick end labeling (TUNEL)-assay and immunofluorescence experiments; counterstaining was performed with 4′-6-diamino-2-phenylindole (DAPI) after the application of phosphate-buffered saline (PBS) or 4, 4′-diisothiocyanatostilbene-2, 2′-disulfonic acid (DIDS) in transversal sections of injured rat spinal cords. Representative images from spinal cord sections 24 hours after injury of (**a**) PBS- and (**b**) DIDS-treated animals stained with TUNEL. (**c**) Graph indicating the number of TUNEL-positive cells in the sections of PBS- and DIDS-treated animals. Representative images from spinal cord sections 24 hours after injury with delineated areas indicating the focus of the lesion and surrounding areas of (**d**) PBS- and (**e**) DIDS-treated animals stained with TUNEL. (**f**) Graph indicating the number of TUNEL-positive cells from injury focus to adjacent areas in the sections of PBS- and DIDS-treated animals. Representative images from spinal cord sections 24 h after injury of (**g**) PBS- and (**h**) DIDS-treated animals stained with anti-oligodendrocyte specific protein (OSP, green), TUNEL (red) and DAPI (blue), with high magnification of the respective selected area. (**i**) Graph indicating the number of OSP-positive cells that also labels with TUNEL. Representative images from spinal cord sections 24 h after injury of (**j**) PBS- and (**k**) DIDS-treated animals stained with anti-neuro-chrome pan neurofilament marker (NCP), TUNEL (red), and DAPI (blue), with high magnification of the respective selected area. (**l**) Graph indicating the number of NCP-positive cells that also label with TUNEL. Bars represent standard error of the mean. Scale bar of a, b, d and e: 250 μm. Scale bar of g, h, j and k: 50 μm. *P < 0.05, **P < 0.01. ^#^*P* < 0.01 in the factor treatment (PBS vs. DIDS) in two-way ANOVA.

In spite of alterations in microglial activation not evidenced with the pharmacological blocker, VDAC1 roles on apoptotic intrinsic pathway were previously described. In order to study the effects of VDAC1 blockade on cell death, we performed a transferase dUTP nick end labeling (TUNEL) assay 24 hours after SCI. We observed that the controls showed a larger number of TUNEL-positive cells compared to the DIDS-treated group (190.6 ± 120 vs. 69.6 ± 43, respectively, P < 0.01). This result agrees with the decrease in spatial apoptosis spread (Fig. 5a–c).

In order to determine the impact of DIDS protective effects in specific cell types, we performed double labeling experiments with TUNEL and anti-oligodendrocyte specific protein (OSP). From the results obtained, we determined that the number of OSP-positive cells that were also labeled with TUNEL in the spinal cord of controls significantly decreased with DIDS treatment (2.83 ± 2.83 vs. 26.5 ± 9.62, respectively, P < 0.05, Fig. 5g–i). We employed a similar approach in double labeling experiments with anti-NCP, a cocktail used to identify different filaments in neurons. We were not able to detect changes in the number of NCP-positive cells that also label TUNEL when comparing controls and the DIDS-treated group (Fig. 5j–l).

### VDAC1 blockade reduces the size of the affected area and increases axonal density

**Figure 6:**
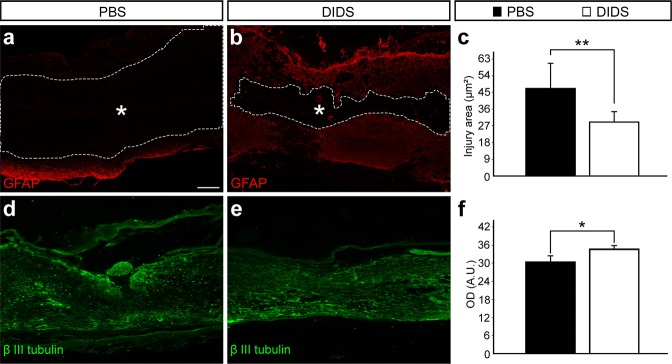
Analysis of the affected area and axonal density of phosphate-buffered saline (PBS) or 4,4′-diisothiocyanatostilbene-2,2′-disulfonic acid (DIDS) sagittal sections 6 weeks after spinal cord injury (SCI). Representative images of the spinal cord sagittal section from (**a**) PBS- and (**b**) DIDS-treated animals stained with anti-glial fibrillary astrocytic antibody (GFAP, red). Dashed lines indicate the GFAP-negative area in the spinal cord after mechanical injury. (**c**) Quantification of the injury area, which corresponds to GFAP-negative area in the spinal cord. Representative images of spinal cord sagittal section from (**d**) PBS- and (**e**) DIDS-treated animals stained with anti – β III tubulin (green). (**f**) Quantification of anti – β III tubulin optical density. Bars represent standard error of the mean. Scale bar: 250μm. *P < 0.05. **P < 0.01.

Since we were able to determine that VDAC1 blockade decreased apoptosis spread, we evaluated possible changes in the affected area and axonal density. GFAP labeling in sagittal sections of the spinal cords revealed that, compared to controls, DIDS-treated animals showed smaller negative area (39.32 ± 12.06 vs. 24.30 ± 4.32, respectively, *P* < 0.01) 6 weeks after the injury (Fig. 6a–c). In order to evaluate axonal density, we performed IF using anti – β III tubulin antibody. It was possible to observe that, compared to the controls, the spinal cord of DIDS-treated animals showed an increase in optical density (30.83 ± 1.76 vs. 33.90 ± 0.07, respectively, *P* < 0.05), which revealed an increase in axonal density 6 weeks after the injury (Fig. 6d–f).

### VDAC1 blockade ameliorates motor recovery

**Figure 7:**
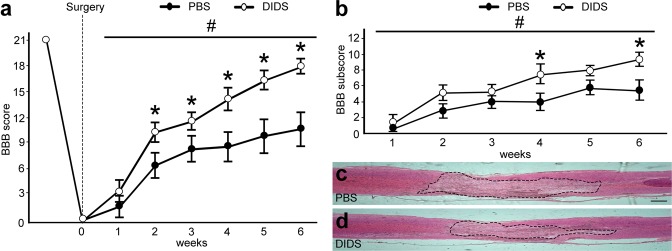
Basso, Beattie, and Bresnahan (BBB) score and motor function recovery after spinal cord injury (SCI) after application of phosphate-buffered saline (PBS) or 4, 4′-diisothiocyanatostilbene-2, 2′-disulfonic acid (DIDS). (**a**) Graph indicating the BBB score of animals that received PBS and DIDS after injury along 6 weeks. (**b**) Graph showing BBB subscore of animals that received PBS and DIDS after injury along 6 weeks. Representative images of spinal cord sagittal sections from (**c**) controls and (**d**) DIDS-treated animals stained with hematoxylin and eosin (HE) after 6 weeks. Dotted line indicates the affected area. Bars represent standard error of the mean. Scale bar: 250 μm. *P < 0.05 compared with the respective time point in the PBS group. ^#^*P* < 0.01 in the factor treatment (PBS vs. DIDS) in two-way ANOVA.

The Basso, Beattie, and Bresnahan (BBB) scale was used 6 weeks after SCI. BBB revealed significant differences in the recovery of the motor function after DIDS pharmacological treatment (Fig. 7a). We were able to observe differences in BBB score 2 weeks after SCI comparing controls and DIDS-treated animals (6.4 ± 1.52 vs. 10.28 ± 1.12, respectively, *P* < 0.05), which was also observed after 3 (8.26 ± 1.57 vs. 11.57 ± 1.01, *P* < 0.05), 4 (8.60 ± 1.69 vs. 14.14 ± 1.36, *P* < 0.05) and 5 (9.8 ± 2.05 vs. 16.35 ± 1.03, *P* < 0.05) weeks. After 6 weeks, PBS animals could sweep their paws without backing up their body, while DIDS-treated animals were able to move normally and reached a higher BBB score (10.64 ± 2.04 vs. 18.00 ± 0.86, respectively, *P* < 0.05, Fig. 7a).

Moreover, we supplemented BBB analyses by sub-scoring the fine details of locomotion. After 4 weeks, when compared to controls, DIDS-treated animals reached higher scores (3.93 ± 0.99 vs. 7.45 ± 0.98, *P* < 0.01), a condition which was not observed after 5 weeks, but was also detected after 6 weeks (5.42 ± 1.18 vs. 9.21 ± 0.79, *P* < 0.01, Fig. 7b). In fact, we were able to observe that, when compared to the controls, DIDS-treated animals showed reduced affected areas in the sagittal sections of spinal cords after 6 weeks of SCI (Fig. 7c,d).

## Discussion

VDAC1 protein forms a channel in the outer mitochondrial membrane, which allows the passage of ATP/ADP between the mitochondrial matrix and cytoplasm. This process is essential for cell metabolism, apoptosis, and ROS generation^50^. It is known that VDAC1 deficiency reduces the cellular production of ATP in VDAC1 knockout mice^51^. Here, we showed that reduced VDAC1 expression levels in spinal cord primary cell cultures caused decrease in mitochondrial metabolism and reduction of neurite growth. It is possible that the absence of ATP impaired the actin polymerization for neurite formation^52^, which is also an important neuronal cell response after mechanical injury. It should be stressed that a more effective knockdown, obtained as previously described^53^, might increase the effects that we observed.

To our knowledge, VDAC1 distribution has been first characterized in the spinal cord cells in the present study, since only WB experiments were performed previously^54,55^. As reported in other parts of the nervous system, this porin was found in several organelles, including the outer mitochondrial membrane^56^, endoplasmic reticulum^57^, cytosol^30^ and cell membrane^58^. Here, we were able to determine that this porin is abundant in both neurons and glial cells from the spinal cord. Previous studies focusing on other parts of the nervous system reported VDAC1 in neurons and glial cells, corroborating with our findings^59^. In the spinal cord of amyotrophic lateral sclerosis mice, VDAC1 and other components of the apoptosis intrinsic pathway were found enriched in motor neurons^60^.

Although VDAC1 protein levels did not alter after SCI, we observed increased IF labeling using anti-VDAC1-PORIN in the ventral horn after injury. We hypothesize that this increase could be related to changes in the association of other proteins with VDAC1. It is known that apoptosis inducers lead to the formation of homo-oligomers and/or hetero-oligomers containing VDAC1 and pro-apoptotic proteins, permitting the release of Cyto-C and triggering the apoptotic cell death^61^. On the other hand, anti-apoptotic proteins such as B-cell lymphoma 2 (Bcl-2) and B-cell lymphoma extra large (Bcl-xL) associate with VDAC1 in physiological conditions^34,35^, therefore dissociation during the progression of the neurodegenerative process might increase the epitope exposition. Additionally, the increase in VDAC1 N-terminal IF labeling could be related to the externalization of the N-terminal region on the presence of apoptotic signals^26^. The N-terminal region of VDAC1 was reported to be involved in the release of pro-apoptotic proteins and apoptosis, while the truncated form of N-terminal promotes apoptosis resistance^62^.

SCI also triggers the spread of pro-inflammatory molecules, which leads to reactive gliosis and microglial activation as major tissue responses. Microglia is able to change its morphology and proteome in response to tissue environment and are normally activated in the course of the inflammation process. After their activation, inflammatory molecules are released, triggering secondary cell death. DIDS is a pharmacological blocker of VDAC1 oligomerization and, according to our results, has no impact on the mitochondrial metabolism. Moreover, the treatment with DIDS did not reverse the number of Iba-1-, CD86- and iNOS-positive cells, nor affected microglia branching, although previous studies demonstrated that DIDS can inhibit the expression and secretion of serum IL1-α and macrophage activation in a sepsis animal model^63^. In addition, the blockade of VDAC1 oligomerization seems to act on astrocyte response following injury, increasing cell hypertrophy rate and glial scar formation as demonstrated through GFAP analysis^64^. The role of reactive astrogliosis and glial scar formation remains controversial concerning axonal regrowth after spinal cord injury. However, it was previously demonstrated a fundamental role in motor function preservation caused by blood-brain barrier reestablishment and regulation of the inflammation process^65^.

Despite VDAC1 participation in tissue response to injury, this porin has specific roles on apoptosis after SCI. Previous studies using staurosporine-induction of apoptosis in salmonid hepatoma and gill cells revealed that DIDS was efficient in inhibiting apoptosis^66^. This finding corroborates with our TUNEL assay results, which showed that DIDS was effective in decreasing cell death after SCI. We hypothesize that DIDS alter the physical conformation of VDAC1, which prevent some molecules responsible for apoptosis from being released by the mitochondria, such as Cyto-c, apoptosis inducing factor, and second mitochondria-derived activator of caspases/direct IAP-binding protein with Low PI (Smac/DIABLO)^66^. It was previously determined that DIDS not only inhibits VDAC1 oligomerization, Cl^−^ fluxes and Cyto-C release, but also acts on caspase activity directly^67^.

Within the first 24 hours after SCI, a massive oligodendrocyte cell death occurs through necrosis and/or apoptosis, leading to primary demyelination of axons at the lesion site^68,69^. The second wave of oligodendrocyte cell death increases the demyelination about 3 weeks after injury and the axons fully lose myelin^70^. The apoptosis spread occurs mostly by Ca^2+^ influx, glutamate excitotoxicity, hypoxia, and free radical formation. These molecules act on first apoptosis signal (FAS) and p75 receptors, which are overexpressed in oligodendrocytes, but not in neurons, after SCI^71^. Interestingly, despite the accumulation of VDAC1 not being specifically high in glial cells, the blockade of VDAC1 oligomerization indisputably decreased oligodendrocyte cell death. On the other hand, neuronal cell death did not decrease due to the VDAC1 oligomerization blockade. This data suggests that oligodendrocytes undergo apoptosis via the intrinsic pathway after SCI, while neurons seem to enter in a cell death program mostly from other cellular pathways.

We observed that VDAC1 oligomerization blockade decreased the number of TUNEL-positive cells mainly in the spinal cord white matter. Prevention of oligodendroglia cell loss probably reflects on processes such as the recovery of both movement initiation and control^72,73^, improved tissue response to Wallerian degeneration processes^73^, axonal remyelination^74^, which is consistent with our results related to the decrease in the tissue cavity, as well as enrichment of locomotor function recovery.

In conclusion, we propose that DIDS prevented oligodendrocyte cell death after SCI without changing microglial activation. Taken together, our results revealed that the blockade VDAC1 oligomerization might be considered for the development of new treatment strategies aimed to ameliorate the consequences of SCI.

## Material and Methods

This study was approved by the Ethics Committee for Animal Experimentation of the Universidade Federal do ABC (protocol 5321200217). Animal manipulations were conducted in accordance with guidelines of the NIH and the Brazilian Society for Laboratory Animals.

### Spinal cord primary cell culture experiments

Neonates (males and females) of wistar rats (*Rattus norvegicus*) 0–3 days postnatal (P0-P3) were used for spinal cord primary cell cultures. The cell culture method was based on previously described studies^75^. The animals were briefly decapitated for the spinal cord dissection. After washing with buffer A (10 μg/ml penicillin/streptomycin), which contained an antibiotic, the tissue was first mechanically dissociated with a scalpel blade, then enzymatically with trypsin 0.15% (Trypsin 2.5% 10x, Thermo Fischer 15090046) at 37 °C for 10 min. Using a Pasteur pipette, the tissue was again dissociated and homogenized. Neurobasal medium (Sigma-Aldrich) containing 10% fetal bovine serum was used for trypsin inhibition. After transferring the samples to a 15 mL conical tube, it was centrifuged for 4 min at 1,510 rpm. After releasing the supernatant, the cells were resuspended and cultured in neurobasal medium with 10% fetal bovine serum, supplemented with 0.1 mM glutamine, 10 μg/ml penicillin/streptomycin, and 2% B27 (Invitrogen/Life Technologies). The cells were cultured in high density at 1 × 105 cells/ml. Cells were maintained in the incubator with 5% CO_2_, 37 °C, and controlled humidity for 14 days *in vitro* for complete cell maturation.

After this period, cell cultures were submitted to VDAC1 MO (Gene Tools, Philomath, OR), which were designed against the mRNA sequence encoding VDAC1 (5′-ATATGTGGGAGGCACAGCCATGTTC-3′). A stock solution of MOs were diluted in autoclaved MilliQ water (1 mM). MOs were delivered to the spinal cord cells in a concentration of 10 μM with 0.6% Endo-Porter as manufactory protocol suggest^76^. In MO experiments, control cell cultures were treated with scramble (SCR) MOs (Gene Tools, Philomath, OR). In parallel, 4,4′-diisothiocyanatostilbene-2,2′-disulfonic acid (DIDS) was delivered to the cell culture in a concentration of 25 μM diluted in PBS. Both treatments were applied to the culture for 48 h. The mitochondrial metabolism was measured using 3-(4,5-dimethylthiazol-2-yl)-2,5-diphenyltetrazolium bromide (MTT tetrazolium reduction assay). The MTT substrate was prepared in a physiologically balanced solution, added to cells in the culture at a final concentration of 0.2–0.5 mg/ml, and incubated for 1 to 4 hours. Formazan concentration (presumably directly proportional to the number of viable cells) was measured at 570 nm absorbance. We employed pure cell medium as negative control (CTL−), DMSO (dimethyl sulfoxide) as positive cell death control (CTL+), and PBS as vehicle control. Results obtained in MTT experiments were submitted to one-way ANOVA followed by Dunnett post-hoc test. Significance level was set at 5%.

### Animal procedures

70 adult male Wistar rats (*Rattus norvegicus*) weighting 300–350 g received 5 mg/k of tramadol hydrochloride i.m. 1 h before anesthesia with 1.5%–2.5% isoflurane for laminectomy and SCI. The injury protocol was adapted from Cristante *et al*.^77^. Spinal cord at T8 level was exposed by laminectomy without visual dura mater injury. The near spine processes were clamped to ensure that the injury procedure and compression were performed during 15 sec using the Impactor (New York University) with 10 g of weight and 12.5 mm of free fall. Muscles and skin were sutured separately. Following surgery, rats received 5 mg/kg pentabiotic i.m. The same dose of tramadol hydrochloride was applied for three days at 8 h intervals. In addition, manual bladder emptying was performed twice a day. All animals were kept on a 12 h light/dark cycle, food and water a*d libitum* with 22 °C ± 2 °C, and 75% relative humidity. For pharmacological treatment and vehicle group, 5 mg/kg of DIDS or PBS were injected i.p. 1 and 24 h after injury.

### Tissue processing

Animals were euthanized with urethane and transcardiac perfused with saline buffer 0.9%, followed by 4% paraformaldehyde (PFA) in 0.1 M phosphate buffer (PB), pH 7.3. A spinal cord segment (N = 3–5) containing the compression injury area and further adjacent segments within 1.5 cm was dissected, cryoprotected in 30% sucrose in 0.1 M PB at 4 °C overnight, and embedded in OCT compound. With regard to WB assays, spinal cord segments (N = 9) from T6-T13 were briefly dissected, washed with PBS, and homogenized in RIPA buffer (50 mM Tris, 150 mM NaCl, 0.1% SDS, 0.5% sodium deoxycholate, 1% Triton X-100, and protease inhibitors). All samples were stored at −80 °C until required.

### Immunofluorescence (IF) assay

Coronal and sagittal sections of spinal cord samples with 12 μm were obtained using a cryostat (Leica, CM1860). The slides were incubated overnight with primary antibodies (Table 1) in a solution containing 5% normal donkey serum (Sigma, D9663) and 0.5% Triton X-100 diluted in PB 0.1 M at room temperature. After serial washes with PB 0.1 M, sections were incubated with 1:500 Alexa488 fluorescent secondary antibody diluted in 0.5% Triton X-100 on PB 0.1 M for two hours at room temperature. The slices were counterstained with 4′-6-diamino-2-phenylindole (DAPI). For double IF, the same protocol was performed using Alexa 546 secondary antibody.

### Western blotting (WB)

Homogenates were centrifuged for 20 min at 14,000 G and 4 °C to remove insoluble material. Protein concentration was determined using the BCA method (Thermo Scientific, Rockford, IL, USA, catalog # 23225), and bovine serum albumin was used as the standard in accordance with the manufacturer’s instructions. Proteins in the membrane preparations were separated using sodium dodecyl sulfate-polyacrylamide gel electrophoresis (SDS-PAGE; 10% gel) and transferred onto nitrocellulose membranes. Blots were incubated with 5% non-fat milk in a Tris-buffered saline with Tween 20 (0.1%) (TBST) buffer for 2 h at room temperature to block nonspecific binding of antibodies. After rinsing the blots with TBST, they were incubated overnight with primary antibodies diluted in TBST/5% non-fat milk. After incubation with primary antibodies (Table 1), blots were rinsed with TBST and incubated with goat anti-rabbit peroxidase (ECLTM kit; Amersham, Buckinghamshire, England) for 2 h at room temperature. For internal control, we used anti – β III tubulin (ab78078). Detection of labeled proteins was achieved using the enhanced chemiluminescent system (ECLTM kit; Amersham). The optical densities (ODs) of the bands were determined using ImageJ software (National Institute of Mental Health, Bethesda, Maryland, USA). Data were entered into Student’s t-test with significance level set at 5%.

### Motor function evaluation

The Basso, Beattie, and Bresnahan (BBB, 1995) locomotor rating scale was performed weekly (N = 14) at the same conditions during 6 weeks after the experimental procedure. The scale ranges from 0 to 21 scores, reflecting the locomotor conditions of the animal, where a score of 0 represents total hindlimb paraplegia, while 21 indicates normal hindlimb activity. In addition, we determined the BBB subscore, which reflects toe clearance, hindlimb rotation, and tail usage regardless of coordination^78^. Data were analyzed using two-way ANOVA followed by pairwise comparisons with Holm-Sidak, with the significance level set at 5%.

### TUNEL assay

Terminal kit Deoxynucleotidyl Transferase (TdT)-mediated dUTP Nick End Labeling (TUNEL) was used for *in situ* detection of apoptosis (Life Technologies, Cat #C10617, C10618, C10619, USA) in spinal cord sections (12 μm) assembled on gelatinized slides. The fixed tissue was washed twice (2 min) in 0.1 M PBS. After drying, TdT Reaction Buffer was added for 10 min, followed by the TdT reaction solution for 1 hour at 37 °C and washed in PBS solution with 3% albumin and 0.1% Triton x-100 for 5 min. The slides were then incubated for 1 hour with Click-iT Plus TUNEL solution prepared according to the manufacturer’s protocol. Finally, the sheets were washed again in the solution made up of BSA 3% and 0.1% Triton x-100. For double labeling experiments, IF was performed after TUNEL assay.

### Hematoxylin and eosin (HE) staining protocol

The spinal cord sections were heated at 45 °C for 40 min, hydrated on a serial dilution of alcohol (100%, 80%, and 70%) for 5 min each, and washed in distilled water for 7 min. Samples were immersed in hematoxylin solution for 3 min and washed in running water for 3 min. Afterwards, the sections were counterstained with eosin solution for 7 min followed by 3 min of running tap water wash. Finally, the samples were dehydrated and washed twice with xylol for 5 and 10 min.

### Image acquisition and analysis

The spinal cord cultures and sections were analyzed under a fluorescence microscope (DM 5500, Leica Microsystems, Germany) coupled to a camera for image capture (DFC 365 FX, Leica Microsystems, Germany). Image analysis was performed with ImageJ software (National Institute of Mental Health, Bethesda, Maryland, USA) and data were exported to Excel (Microsoft, Redmond, WA, USA) or SigmaStat 3.5 (Systat Software, San Jose, CA, USA) for respective statistical analysis with 5% significance level. Images and charts were prepared with Adobe Photoshop CS5 (Adobe Systems Inc., San Jose, CA, USA).

#### VDAC1 MO and DIDS treatment in spinal cord primary culture

The neurite length and the radius of the cell were measured using the Synapse Detection (SynD) toolbox to MATLAB [1], which aims to detect cell morphological characteristics. It was done in two steps: 1) The soma was detected by manual selection to ensure that the whole soma was considered with small adjustments. 2) The neurites were detected by semi-automatic selection in which it is possible to select detection threshold and filter size parameters. The filter size represents the width of the neurites. For each image, the adjustment of the filter size was done manually, with values ranging from 1.3 to 2 micrometers. After automatic detection, manual adjustments were made to capture neurites not identified by the SynD. At the end of soma and neurite detection, SynD generates a mask containing the pixels where the neurites are present. The length was calculated using the automatic tool of SynD that realizes the skeletizing of neurites, then calculating the length in micrometers. The radius of the cell was defined as the circle centered in the soma and which is capable of circumscribing all the neurites. Although these measures were subjected to manual choice, the results obtained in the analysis were robust to change in parameters such as detection threshold and filter size. Both parameters, length and radius, were exported. Data were entered into Student’s t-test with significance level set at 5%.

#### Double labeling IF

Three sections in five slides of each group were selected using deconvoluted images with 40x magnification. Pixel intensity profile was obtained using ICY software^79^.

#### VDAC1 PORIN and N-term mean gray value

Mean gray value was obtained using ImageJ Measure tool, where gray values of all the pixels in the selection were divided by the number of pixels detected. Data were entered into Student’s t-test with significance level set at 5%.

#### Quantification of CD86 - positive cells

Double-blind manual counting was performed to evaluate the number of CD86-positive cells. CD86 surrounding and overlap nucleus were considered a positive cell. Data were exported and statistical analysis was performed using Student T-Test with 5% significance.

#### Microglial branching

As previously reported^80^, microglial branching was analyzed based on Iba-1 labeling of 24 different cells on both spinal cord horns and AnalyzeSkeleton plugin in ImageJ. Data were entered into Student’s t-test with significance level set at 5%.

#### Quantification of iNOS - positive cells

Double-blind manual counting was performed to evaluate the number of iNOS-positive cells. iNOS surrounding and overlap nucleus were considered a positive cell. Data were entered into Student’s t-test with significance level set at 5%.

#### Quantification of TUNEL - positive cells

TUNEL-positive cells on spinal cord were determined through “TUNEL cell counter-master” plugin^76^. Parameters were set for minimum and maximum cell area as 1 and 25, respectively, and all images were analyzed. Data were entered into Student’s t-test with significance level set at 5%.

#### Radial analyses of TUNEL - positive cells distribution from the injury epicenter

TUNEL – positive cells were submitted to a radial analysis in order to determine the apoptosis spread after SCI. For this, circles were generated with 250 μm as the initial diameter and each new circle had an increase of 250 μm. Prior to image analysis, the injury epicenter was delimited in the center of dorsal horns of each image. “TUNEL cell counter-master” plugin was used to identify and count the TUNEL-positive cells. After that, the number of cells per circle were counted in blind analysis. The data were entered in ANOVA two-way with the post-test Holm-Sidak and significance level set at 5%.

#### Affected area labeled by GFAP

The cavity area was determined by the absence of GFAP labeling in the spinal cord. Data were entered into Student’s t-test with significance level set at 5%.

#### Anti – β III tubulin density

The optical densities (ODs) of the images were determined using ImageJ software (National Institute of Mental Health, Bethesda, Maryland, USA). Data were entered into Student’s t-test with significance level set at 5%.

**Table 1:** Antibodies used in this study.

Protein	Antibody	Antibody Concentration
VDAC1 N-term	Abcam/catalog # AB72181	IF – 1:100 WB – 1:1000
VDAC1 PORIN	Abcam/catalog # AB103884	IF – 1:100 WB – 1:1000
β III tubulin	Abcam/catalog # ab78078	IF – 1:200
choline acetyltransferase (ChAT)	Millipore/catalog # AB144P	IF – 1:50
glial fibrillary acidic protein (GFAP)	Sigma/catalog # G3893	IF – 1:200
calretinin (CR)	Millipore/catalog # MAB1568	IF – 1:200
calbindin (CB)	Sigma-Aldrich/catalog # C9848	IF – 1:200
inducible nitric oxide synthase (iNOS)	Sigma-Aldrich/catalog # N7782	IF – 1:200
ionized calcium-binding adapter molecule 1 (Iba-1)	Wako/catalog # 019-19741	IF - 1:200
oligodendrocyte specific protein (OSP)	Abcam/catalog # ab53041	IF - 1:200
neuro-chrome pan (NCP - neurofilament marker)	Millipore/catalog # MAB2300	IF - 1:200
CD86	Abcam/catalog # ab125028	IF – 1:200

## References

[1] Kigerl KA, Popovich PG (2009). Toll-like receptors in spinal cord injury. Current topics in microbiology and immunology.

[2] Taoka Y, Okajima K (1998). Spinal cord injury in the rat. Progress in neurobiology.

[3] Tator CH, Fehlings MG (1991). Review of the secondary injury theory of acute spinal cord trauma with emphasis on vascular mechanisms. Journal of neurosurgery.

[4] Paschon V, Higa GS, Resende RR, Britto LR, Kihara AH (2012). Blocking of Connexin-Mediated Communication Promotes Neuroprotection during Acute Degeneration Induced by Mechanical Trauma. PLoS One.

[5] Paschon V (2013). A new and reliable guide for studies of neuronal loss based on focal lesions and combinations of *in vivo* and *in vitro* approaches. PloS one.

[6] Wang HF (2017). Effect of glial cells on remyelination after spinal cord injury. Neural regeneration research.

[7] Sandler AN, Tator CH (1976). Effect of acute spinal cord compression injury on regional spinal cord blood flow in primates. Journal of neurosurgery.

[8] Collins WF (1983). A review and update of experiment and clinical studies of spinal cord injury. Paraplegia.

[9] Beattie MS, Hermann GE, Rogers RC, Bresnahan JC (2002). Cell death in models of spinal cord injury. Progress in brain research.

[10] Hall ED, Yonkers PA (1989). Mechanisms of neuronal degeneration secondary to central nervous system trauma or ischemia. J Neurotrauma.

[11] Agrawal SK, Fehlings MG (1996). Mechanisms of secondary injury to spinal cord axons *in vitro*: role of Na+, Na(+)-K(+)-ATPase, the Na(+)-H+ exchanger, and the Na(+)-Ca2+ exchanger. The Journal of neuroscience: the official journal of the Society for Neuroscience.

[12] Schwartz G, Fehlings MG (2002). Secondary injury mechanisms of spinal cord trauma: a novel therapeutic approach for the management of secondary pathophysiology with the sodium channel blocker riluzole. Progress in brain research.

[13] Kuroiwa M (2014). Effect of amiloride on endoplasmic reticulum stress response in the injured spinal cord of rats. The European journal of neuroscience.

[14] Almad A, Sahinkaya FR, McTigue DM (2011). Oligodendrocyte fate after spinal cord injury. Neurotherapeutics: the journal of the American Society for Experimental NeuroTherapeutics.

[15] Basso DM (1996). MASCIS evaluation of open field locomotor scores: effects of experience and teamwork on reliability. Multicenter Animal Spinal Cord Injury Study. Journal of neurotrauma.

[16] van Niekerk EA, Tuszynski MH, Lu P, Dulin JN (2016). Molecular and Cellular Mechanisms of Axonal Regeneration After Spinal Cord. Injury. Molecular & cellular proteomics: MCP.

[17] Aouacheria A (2017). Connecting mitochondrial dynamics and life-or-death events via Bcl-2 family proteins. Neurochemistry international.

[18] Manczak M, Sheiko T, Craigen WJ, Reddy PH (2013). Reduced VDAC1 protects against Alzheimer’s disease, mitochondria, and synaptic deficiencies. Journal of Alzheimer’s disease: JAD.

[19] Jacobs D (2019). Probing Membrane Association of alpha-Synuclein Domains with VDAC Nanopore Reveals Unexpected Binding Pattern. Scientific reports.

[20] Saminathan A, Vinoth KJ, Low HH, Cao T, Meikle MC (2013). Engineering three-dimensional constructs of the periodontal ligament in hyaluronan-gelatin hydrogel films and a mechanically active environment. Journal of periodontal research.

[21] Shoshan-Barmatz V, Ben-Hail D (2012). VDAC, a multi-functional mitochondrial protein as a pharmacological target. Mitochondrion.

[22] Elmore S (2007). Apoptosis: a review of programmed cell death. Toxicologic pathology.

[23] Wang X (2001). The expanding role of mitochondria in apoptosis. Genes & development.

[24] Khosravi-Far R, Esposti MD (2004). Death receptor signals to mitochondria. Cancer biology & therapy.

[25] Ghosh T, Pandey N, Maitra A, Brahmachari SK, Pillai B (2007). A role for voltage-dependent anion channel Vdac1 in polyglutamine-mediated neuronal cell death. PloS one.

[26] Shoshan-Barmatz V (2010). VDAC, a multi-functional mitochondrial protein regulating cell life and death. Molecular aspects of medicine.

[27] Shoshan-Barmatz V, Mizrachi D (2012). VDAC1: from structure to cancer therapy. Frontiers in oncology.

[28] Marginedas-Freixa I (2018). Human erythrocytes release ATP by a novel pathway involving VDAC oligomerization independent of pannexin-1. Scientific reports.

[29] Pavlov E (2005). The mitochondrial channel VDAC has a cation-selective open state. Biochimica et biophysica acta.

[30] Shoshan-Barmatz V, Israelson A, Brdiczka D, Sheu SS (2006). The voltage-dependent anion channel (VDAC): function in intracellular signalling, cell life and cell death. Current pharmaceutical design.

[31] Bayrhuber M (2008). Structure of the human voltage-dependent anion channel. Proceedings of the National Academy of Sciences of the United States of America.

[32] Azoulay-Zohar H, Israelson A, Abu-Hamad S, Shoshan-Barmatz V (2004). In self-defence: hexokinase promotes voltage-dependent anion channel closure and prevents mitochondria-mediated apoptotic cell death. The Biochemical journal.

[33] Zalk R, Israelson A, Garty ES, Azoulay-Zohar H, Shoshan-Barmatz V (2005). Oligomeric states of the voltage-dependent anion channel and cytochrome c release from mitochondria. The Biochemical journal.

[34] Arbel N, Ben-Hail D, Shoshan-Barmatz V (2012). Mediation of the antiapoptotic activity of Bcl-xL protein upon interaction with VDAC1 protein. J Biol Chem.

[35] Arbel N, Shoshan-Barmatz V (2010). Voltage-dependent anion channel 1-based peptides interact with Bcl-2 to prevent antiapoptotic activity. J Biol Chem.

[36] Raghavan A, Sheiko T, Graham BH, Craigen WJ (2012). Voltage-dependant anion channels: novel insights into isoform function through genetic models. Biochimica et biophysica acta.

[37] Caterino M (2017). Protein-protein interaction networks as a new perspective to evaluate distinct functional roles of voltage-dependent anion channel isoforms. Molecular bioSystems.

[38] De Pinto V (2003). New functions of an old protein: the eukaryotic porin or voltage dependent anion selective channel (VDAC). The Italian journal of biochemistry.

[39] Mannella CA (1997). Minireview: on the structure and gating mechanism of the mitochondrial channel, VDAC. Journal of bioenergetics and biomembranes.

[40] Pfaller R, Freitag H, Harmey MA, Benz R, Neupert W (1985). A water-soluble form of porin from the mitochondrial outer membrane of Neurospora crassa. Properties and relationship to the biosynthetic precursor form. The Journal of biological chemistry.

[41] De Stefani D (2012). VDAC1 selectively transfers apoptotic Ca2+ signals to mitochondria. Cell death and differentiation.

[42] Shoshan-Barmatz V (1996). VDAC/porin is present in sarcoplasmic reticulum from skeletal muscle. FEBS Lett.

[43] Thinnes FP (1994). Channel active mammalian porin, purified from crude membrane fractions of human B lymphocytes or bovine skeletal muscle, reversibly binds the stilbene-disulfonate group of the chloride channel blocker DIDS. Biol Chem Hoppe Seyler.

[44] Wohlrab D, Wohlrab J, Markwardt F (2000). Electrophysiological characterization of human keratinocytes using the patch-clamp technique. Exp Dermatol.

[45] Amphoux-Fazekas T (1998). DIDS (4,4′-diisothiocyanatostilbene-2,2′-disulfonic acid) increases iodide trapping, inhibits thyroperoxidase and antagonizes the TSH-induced apical iodide efflux in porcine thyroid cells. Mol Cell Endocrinol.

[46] Cabantchik ZI, Greger R (1992). Chemical probes for anion transporters of mammalian cell membranes. Am J Physiol.

[47] Tomaskova Z, Gaburjakova J, Brezova A, Gaburjakova M (2007). Inhibition of anion channels derived from mitochondrial membranes of the rat heart by stilbene disulfonate–DIDS. J Bioenerg Biomembr.

[48] Wilson MC, Meredith D, Bunnun C, Sessions RB, Halestrap AP (2009). Studies on the DIDS-binding site of monocarboxylate transporter 1 suggest a homology model of the open conformation and a plausible translocation cycle. J Biol Chem.

[49] Keinan N, Tyomkin D, Shoshan-Barmatz V (2010). Oligomerization of the mitochondrial protein voltage-dependent anion channel is coupled to the induction of apoptosis. Mol Cell Biol.

[50] Huang H, Shah K, Bradbury NA, Li C, White C (2014). Mcl-1 promotes lung cancer cell migration by directly interacting with VDAC to increase mitochondrial Ca2+ uptake and reactive oxygen species generation. Cell death & disease.

[51] Okada SF (2004). Voltage-dependent anion channel-1 (VDAC-1) contributes to ATP release and cell volume regulation in murine cells. The Journal of general physiology.

[52] Bernstein BW, Bamburg JR (2003). Actin-ATP hydrolysis is a major energy drain for neurons. The Journal of neuroscience: the official journal of the Society for Neuroscience.

[53] Arif T, Vasilkovsky L, Refaely Y, Konson A, Shoshan-Barmatz V (2017). Silencing VDAC1 Expression by siRNA Inhibits Cancer Cell Proliferation and Tumor Growth *In Vivo*. Mol Ther Nucleic Acids.

[54] Israelson A (2010). Misfolded mutant SOD1 directly inhibits VDAC1 conductance in a mouse model of inherited ALS. Neuron.

[55] Miller AD (2012). Acute traumatic spinal cord injury induces glial activation in the cynomolgus macaque (Macaca fascicularis). Journal of medical primatology.

[56] Magri A (2016). Hexokinase I N-terminal based peptide prevents the VDAC1-SOD1 G93A interaction and re-establishes ALS cell viability. Scientific reports.

[57] Camara AKS, Zhou Y, Wen PC, Tajkhorshid E, Kwok WM (2017). Mitochondrial VDAC1: A Key Gatekeeper as Potential Therapeutic Target. Frontiers in physiology.

[58] De Pinto V, Messina A, Lane DJ, Lawen A (2010). Voltage-dependent anion-selective channel (VDAC) in the plasma membrane. FEBS letters.

[59] McEnery MW (1993). Mitochondrial voltage-dependent anion channel. Immunochemical and immunohistochemical characterization in rat brain. The Journal of biological chemistry.

[60] Martin LJ (2009). The mitochondrial permeability transition pore in motor neurons: involvement in the pathobiology of ALS mice. Experimental neurology.

[61] Li H (2016). Voltage-Dependent Anion Channel 1(VDAC1) Participates the Apoptosis of the Mitochondrial Dysfunction in Desminopathy. PloS one.

[62] Shoshan-Barmatz V, Keinan N, Abu-Hamad S, Tyomkin D, Aram L (2010). Apoptosis is regulated by the VDAC1 N-terminal region and by VDAC oligomerization: release of cytochrome c, AIF and Smac/Diablo. Biochimica et biophysica acta.

[63] Xiang NL (2016). Abrogating ClC-3 Inhibits LPS-induced Inflammation via Blocking the TLR4/NF-kappaB Pathway. Scientific reports.

[64] Banati RB (2004). Mitochondria in activated microglia *in vitro*. Journal of neurocytology.

[65] Silver J, Miller JH (2004). Regeneration beyond the glial scar. Nature reviews. Neuroscience.

[66] Faulkner JR (2004). Reactive astrocytes protect tissue and preserve function after spinal cord injury. The Journal of neuroscience: the official journal of the Society for Neuroscience.

[67] Krumschnabel G, Maehr T, Nawaz M, Schwarzbaum PJ, Manzl C (2007). Staurosporine-induced cell death in salmonid cells: the role of apoptotic volume decrease, ion fluxes and MAP kinase signaling. Apoptosis: an international journal on programmed cell death.

[68] Benitez-Rangel E, Lopez-Mendez MC, Garcia L, Guerrero-Hernandez A (2015). DIDS (4,4′-Diisothiocyanatostilbene-2,2′-disulfonate) directly inhibits caspase activity in HeLa cell lysates. Cell death discovery.

[69] Emery E (1998). Apoptosis after traumatic human spinal cord injury. Journal of neurosurgery.

[70] Norenberg MD, Smith J, Marcillo A (2004). The pathology of human spinal cord injury: defining the problems. Journal of neurotrauma.

[71] Griffiths IR, McCulloch MC (1983). Nerve fibres in spinal cord impact injuries. Part 1. Changes in the myelin sheath during the initial 5 weeks. Journal of the neurological sciences.

[72] Armstrong DM (1986). Supraspinal contributions to the initiation and control of locomotion in the cat. Progress in neurobiology.

[73] Morris R, Whishaw IQ (2016). A Proposal for a Rat Model of Spinal Cord Injury Featuring the Rubrospinal Tract and its Contributions to Locomotion and Skilled Hand Movement. Frontiers in neuroscience.

[74] Cook RD, Wisniewski HM (1973). The role of oligodendroglia and astroglia in Wallerian degeneration of the optic nerve. Brain research.

[75] Langlois, S. D., Morin, S., Yam, P. T. & Charron, F. Dissection and culture of commissural neurons from embryonic spinal cord. *Journal of visualized experiments: JoVE*, 10.3791/1773 (2010). 10.3791/1773 PMC3152854 20505653

[76] Maidana DE (2015). A Novel ImageJ Macro for Automated Cell Death Quantitation in the Retina. Investigative ophthalmology & visual science.

[77] Cristante AF, Barros Filho TE, Marcon RM, Letaif OB, Rocha ID (2012). Therapeutic approaches for spinal cord injury. Clinics.

[78] DePaul MA (2015). Intravenous multipotent adult progenitor cell treatment decreases inflammation leading to functional recovery following spinal cord injury. Scientific reports.

[79] de Chaumont F (2012). Icy: an open bioimage informatics platform for extended reproducible research. Nature methods.

[80] Morrison H, Young K, Qureshi M, Rowe RK, Lifshitz J (2017). Quantitative microglia analyses reveal diverse morphologic responses in the rat cortex after diffuse brain injury. Scientific reports.

